# Thermodynamics of camphor migration in cytochrome P450cam by atomistic simulations

**DOI:** 10.1038/s41598-017-07993-0

**Published:** 2017-08-10

**Authors:** J. Rydzewski, W. Nowak

**Affiliations:** Institute of Physics, Faculty of Physics, Astronomy and Informatics, Nicolaus Copernicus University, Grudziadzka 5, 87-100 Torun, Poland

## Abstract

Understanding the mechanisms of ligand binding to enzymes is of paramount importance for the design of new drugs. Here, we report on the use of a novel biased molecular dynamics (MD) methodology to study the mechanism of camphor binding to cytochrome P450cam. Microsecond-long MD simulations allowed us to observe reaction coordinates characterizing ligand diffusion from the active site of cytochrome P450cam to solvent via three egress routes. These atomistic simulations were used to estimate thermodynamic quantities along the reaction coordinates and indicate diverse binding configurations. The results suggest that the diffusion of camphor along the pathway near the substrate recognition site (SRS) is thermodynamically preferred. In addition, we show that the diffusion near the SRS is triggered by a transition from a heterogeneous collection of closed ligand-bound conformers to the basin comprising the open conformations of cytochrome P450cam. The conformational change accompanying this switch is characterized by the retraction of the F and G helices and the disorder of the B′ helix. These results are corroborated by experimental studies and provide detailed insight into ligand binding and conformational behavior of the cytochrome family. The presented methodology is general and can be applied to other ligand-protein systems.

## Introduction

Cytochrome P450 enzymes are ubiquitous monooxygenases that contain heme^1^. They are responsible for a variety of life processes including carcinogenesis and drug metabolism as well as the degradation of xenobiotics and biosythesis of numerous natural products^2, 3^. Remarkably, 11 cytochrome P450 enzymes contribute to the biotransformation of 70% of clinically used drugs^4, 5^, which makes cytochrome P450 enzymes a potential template to produce biotechnologically relevant chemicals such as an antimalarial drug artemisinin, or statins^6^. The most well understood cytochrome P450cam from soil bacterium *Pseudomonas putida* catalyzes the hydroxylation of camphor (its natural substrate) to 5-hydroxy-camphor. This enzyme has been used as a model for many cytochromes P450 and was the first cytochrome P450 protein structure solved by X-ray crystallography^7^. The active site of cytochromes P450 is situated deep inside the protein matrix on the distal site of the heme prosthetic group, rather than exposed on the protein surface^8^. Thus, the crystal structures of substrate-bound cytochrome P450cam do not show any obvious access or exit channels along which ligands could migrate. Consequently, the enzyme specificity, thermodynamics and conformational states can be influenced by how substrates pass through multiple channels of the protein to access the active site and how products escape toward solvent.

In the proteins with no clear ligand transport pathways, ligand diffusion is challenging to model computationally, because the entrance and egress trajectories of the ligand generally involve migration through binding intermediates linking the dissociated and associated states^9, 10^. Furthermore, ligand diffusion is difficult to assess also experimentally due to the dynamical complexity of protein structures, and in the virtual absence of time-resolved crystallography experiments on ligand intermediates, the actual ligand expulsion pathways remain undetermined^11^. And yet, detailed knowledge of binding and release mechanisms is fundamental for the comprehension of the biological function of enzymes. From a computational viewpoint, the main complication arises from two reasons. First, the reaction coordinate for a pathway that interpolates the transition between the bound and unbound state is difficult to assess. Second, the process is not at equilibrium. To sample ligand diffusion within timescales accessible in MD simulations computing nonequilibrium trajectories is necessary since ligand binding occurs on the microsecond-millisecond time scale^12^. Thus, the ligand diffusion and its escape rates are accelerated, making it difficult to estimate thermodynamic and kinetic properties from the simulations. Because of that, several methods have been proposed to tackle the ligand diffusion problem, ranging from steered MD^13^ and its variants^14–16^ through locally enhanced sampling^17–19^, metadynamics^20–22^ and, recently, the memetic sampling (MS)^9, 23, 24^.

Due to their significance, the ligand diffusion pathways have been identified previously and mechanisms of enzymatic trafficking have been investigated in various cytochromes. These studies have provided valuable insights into the ligand diffusion processes in cytochrome P450 enzymes^15, 16, 25–31^. Recently, we have introduced the MS method capable of sampling the ligand diffusion pathways, in which the process of ligand diffusion from a protein is reconstructed by efficient conformational space sampling of the protein matrix in such a way that the obtained egress trajectories minimize effective interaction energy between the ligand and protein^23, 24^. In MS a biasing force with a direction determined by this minimization process is used to guide a ligand along a curved egress route. With MS one can calculate reaction coordinates of ligand diffusion which atomistically describe transitions of a ligand between bound and unbound states. Our simulations revealed three essential classes of the camphor diffusion trajectories in cytochrome P450cam. They were named pathways pw1, pw2 and pw3. The migration of camphor along pw1 involves passing between the I helix, known for its small thermal motions, and the distal side of the heme. The most frequent pathway (pw2) engages the region involving migration near the SRS site that is variable in sequence and structure between different cytochromes in the P450 family^32^. Its high flexibility in all P450s suggests a role in selectivity of a substrate. There are theoretical results suggesting that pw2 is preferred by substrates^15, 16, 26, 32^. Along pw3 camphor diffuses from the active site to the proximal side of heme, near the C and L helices. Despite this, transport along the pathways remains poorly understood, perhaps due to the absence of exhaustive sampling of the cytochrome configuration space that is crucial to compute thermodynamic quantities.

In what follows, we continued the research by aiming at understanding how the egress pathways and the fluctuations of the residues of cytochrome P450cam active in the transport contribute to the egress pathway preference and its thermodynamics. We addressed the camphor preference for each ligand diffusion pathway. In order to achieve this goal, we used an unprecedented combination of enhanced sampling methods, including calculating the reaction coordinates of ligand diffusion on-the-fly during MD simulations and metadynamics. To the best of our knowledge, we calculated for the first time the free energy along the previously identified camphor diffusion pathways. Combined with our previous studies of the camphor migration within the matrix of cytochrome P450cam and searching for escape via diffusion pathways, we obtained a full quantitative description of the camphor migration from the active site including structural and thermodynamic information.

## Results

The present investigation builds on our prior studies of ligand diffusion in cytochrome P450cam and focuses on recovery of free-energy barriers along the identified camphor pathways and structural changes in the enzyme accompanying diffusion (cf. Methods).

### Mechanism of the camphor diffusion in cytochrome P450cam

**Figure 1:**
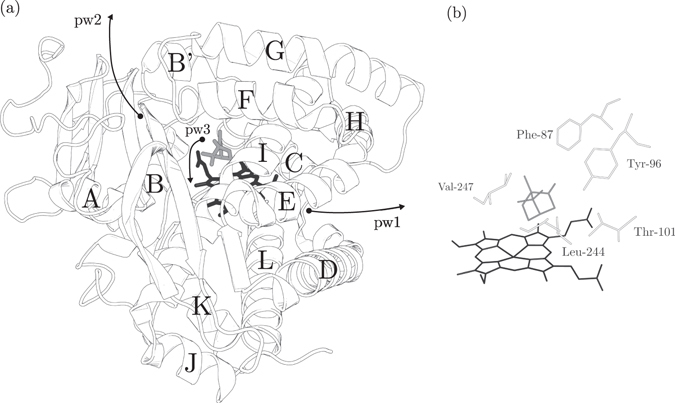
(**a**) Structure of cytochrome P450cam with camphor (gray) and heme (black). Helices are labeled A, B, B′ and C–L. The diffusion exits of pw1-3 are indicated by arrows. (**b**) The active site with camphor at the crystallographic pose and the aligning residues in the distal heme moiety. For a detailed picture of the camphor diffusion from cytochrome P450cam, see Fig. S1 in the Supplementary Information.

The crystal structure of cytochrome P450cam has 13 *α*-helices (A, B, B′ and C–L) and five *β*-sheets (*β*1–*β*5) and it is shown in Fig. 1. We used terminology introduced by Poulos *et al*. in the article describing the crystallographic camphor-bound structure of cytochrome P450cam (PDB ID: 2CPP)^33^. The active site of cytochrome P450cam is located above the proximal helix (L) at the level of the distal helix (I). The initial position of camphor in all the metadynamics simulations is aligned by the B′ helix (Tyr-96), B-B′ loop (Phe-87) and I helix (Val-247 and Leu-244).

During the initial steps of the simulations the keto group of camphor accepts a hydrogen bond from the Tyr-96 hydroxyl group, which controls the position of camphor in the distal heme pocket. Camphor initiates its migration by an adjustment of the Tyr-96 hydroxyl group–hydrogen–camphor carbonyl oxygen angle. This change occurs on all the diffusion pathways, suggesting that it is a preferred position of camphor in the docking pocket. We run 10 ns unbiased MD simulations starting from this pose on each pathway confirming its thermodynamic stability (Table S2). Comparing to the crystallographic pose^33^, the orientation of camphor toward the heme moiety is preserved although the distance between the Tyr-96 hydroxyl group oxygen and the carbonyl oxygen of camphor decreases from 2.9 Å to 2.5 Å, allowing camphor to closely interact with the B′ helix in the active site. Frequently during the camphor diffusion, Tyr-96 adopted a new rotamer conformation, pointing out of the active site and vacating a space for the migrating camphor in the active site. This observation is corroborated by experimental studies in which Tyr-96 was shown to serve as a gateway to substrate binding and migration in the access channel^34^. After the initial stage, camphor adopts a stable pose in the free-energy minimum. At this position, the residues from the relatively short B′ helix (7 residues from 89 to 96) provide a mechanism of selective adjustment to camphor, contributing to free energy and substrate specificity. Next, the camphor diffusion leading to the dissociated state of camphor and cytochrome P450cam starts. The transition between the bound and dissociated states occurs through the multiple pathways facilitated by a number of nonnative transient hydrogen bonds, thus providing selective gating mechanisms.

#### pw1

**Figure 2:**
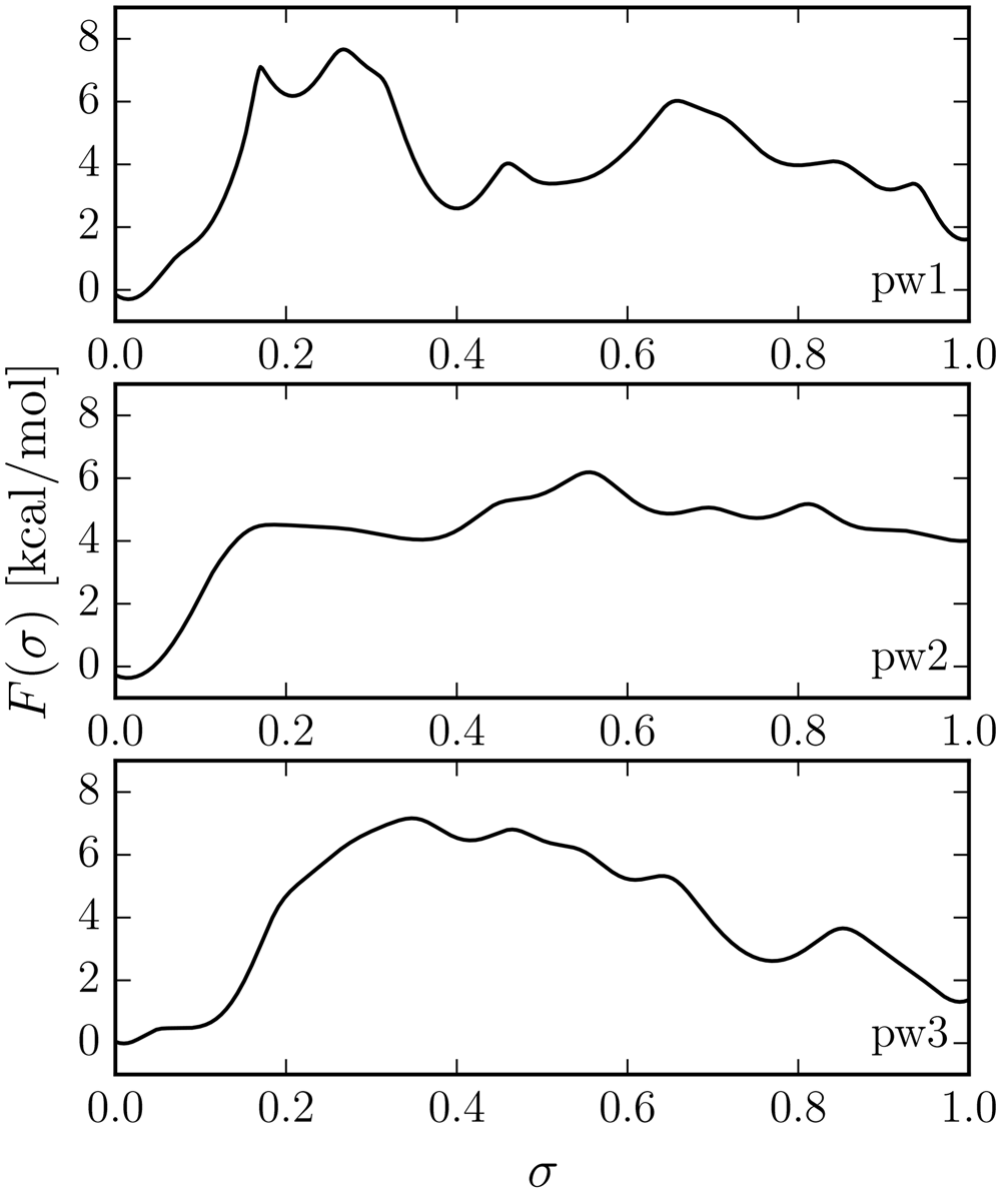
Free-energy profiles *F*(*σ*) along the camphor diffusion pathways pw1-3. [The order parameter *σ* (cf. Methods) is introduced by scaling *s*].

The free-energy profiles along the camphor diffusion pathways pw1-3 are shown in Fig. 2. The migration of camphor along pw1 involves overcoming a region of small thermal motions. First, the camphor diffusion starts with an adjustment toward the I helix and the distal side of the heme group. The existence of the local maxima along pw1 was confirmed by running 50 short unbiased MD simulations for each maximum to check whether camphor diffuses to the neighboring local stable basins (Table S2). The highest free-energy barrier (≈8 kcal/mol at $\sigma \in \mathrm{\{0.17,\; 0.3\}}$) comes from the passing of camphor between the I helix and heme and deforming Cys-242, Leu-245, Leu-246 and Val-247 (Table S1). Although the free-energy curve shows a shallow minimum (*σ* ≈ 0.2), which may indicate a stable intermediate, we run a 10 ns unbiased MD simulation from this collective variable (CV) point and came to a conclusion that camphor migrates back to the active site. This feature in free energy is probably due to a relatively large error of free energy, which is the highest (<1.2 kcal/mol, Fig. S6) in this CV range. After passing below the I helix camphor establishes a stable pose close to the ﻿C, D and E﻿ helices, which was confirmed by 10 ns unbiased MD simulation ($\sigma =0.45\pm 0.05$). The next free-energy local maximum (≈6 kcal/mol at *σ* ≈ 0.66) is related to pushing past the D helix (Val-123, Lys-126, Leu-127 and Arg-130) at the verge of the enzyme structure. Next, camphor diffuses to solvent which is indicated by decreasing free-energy values. Although pw1 was suggested as a potential ligand passage in several articles^15, 16, 23, 24^, we found that the free-energy barrier occurring on this path is the highest (≈8 kcal/mol) in comparison to pw2 and pw3 (≈6 kcal/mol and ≈7 kcal/mol respectively), which makes the migration along pw1 less favorable thermodynamically for a ligand as bulky as camphor.

#### pw2

The diffusion of camphor along pw2 incorporates migration through the SRS near the F/G loop, BC loop, B′ helix and the *β*_1_ strand. The SRS site is conserved in various cytochromes from the P450 family, showing evidence for substrate specificity^35^. The mechanism of egress along pw2 leads to the free-energy barrier (≈4.5 kcal/mol) which has the lowest value to escape from the distal side of heme comparing to pw1 and pw3 (Fig. 2). This fact indicates a preference in choosing pw2 over the other pathways. Metadynamics simulations indicate that the highest free-energy value along pw2 comes from perturbing Tyr-96 and Phe-87 from the B′ helix (Table S1), whose aromatic side-chains gate the camphor migration (≈4.5 kcal/mol at *σ* ≈ 0.17). These findings are in agreement with Winn *et al*.^32^, showing that the side-chain torsions of Phe-87 and Phe-193 contribute to the camphor transport along pw2. This ratcheting/gating mechanism is thus twofold. First, the side-chains of the B′ helix and B-B′ loop provide steric barriers along pw2, and second, donor a hydrogen bond (Tyr-96) to the keto group of camphor.

On the other hand, the camphor egress along pw2 to solvent seems to be more complex than previously described. Namely, after reaching the free-energy maximum, camphor moves toward Ile-395 and Phe-193, and Thr-192 which also donates a hydrogen bond to camphor (Table S1). Before camphor exits to solvent, it is thermodynamically stabilized in a metastable state guarded by Tyr-29, which acts similar to the gates in the active site. From the above picture, one can see that Tyr-69, Phe-87 (*σ* ≈ 0.2, an intermediate state for <37 ps) and Tyr-29 (*σ* ≈ 0.84, a transition point for <578 ps) provide surprisingly complex gating mechanism. Namely, after camphor moves past Tyr-29, the side-chain of Tyr-29 rotates toward the leaving camphor, thus recurring on the diffusion pathway. This ratcheting movement suggests that the described gating mechanism is bidirectional. The values of free energy near *σ* ≈ 1 are high comparing to the other diffusion pathways, since Asp-188, Thr-192, Glu-91, Asn-30 and Pro-31 located near the exit of pw2 are in close proximity of each other, creating a narrow gauge, and thus prohibiting camphor from leaving. At this value of *σ* camphor is still interacting with the neighboring amino acids of cytochrome P450cam.

The results show that salt link tethering plays a role in the preservation of the diffusion pathway. Namely, salt links stabilize the enzyme structure in the proximity of the camphor migration pathway, mostly on the helix-rich side of cytochrome P450cam, Asp-97–Lys-197, Asp-188–Lys-392 and Asp-251–Arg-186. The salt links loosen occasionally while disrupted by the migrating camphor, but relax to their initial positions after camphor moves toward the exit. We observed that the residues that participate in creating salt links establish three patches along pw2. The Asp-251–Arg-186 salt link is created between the I helix and F-G loop in close proximity to the active site. Next, the Asp-97–Lys-197 salt link stabilizes pw2 near the B′-C loop. Perhaps the most interesting is the Asp-188–Lys-392 salt link which is located near the exit route to solvent. It is the least deviated patch during diffusion (the mean distance of the salt link is ≈3.2 Å) that contributes to the formation of the narrow gauge at the exit.

Although there are calculations showing that the transport along pw2 may be divided into several subclasses depending on the exit chosen by camphor^35^, during our metadynamics simulations we do not observe any tendency to diverge from the exit point of the reaction pathway. We suppose that this is strictly related to the algorithm used to uncover reaction coordinates. For instance, several pathways similar to pw2 (pw2a-c) were determined using random acceleration MD (in which camphor was accelerated in a purely random direction)^15^, which did not identify optimal reaction coordinates. Moreover, metadynamics simulations converge to the minimum free-energy pathway, thus providing direct quantitative values of free energy along the pathway.

#### pw3

The transport along pw3 begins from the adjustment of the camphor pose, as described in the case of pw1 and pw2. Subsequently, the side-chain of Tyr-96 rotates in the direction of Phe-87 causing an increase in free energy (≈7.2 kcal/mol). After Tyr-96 vacates a space for the movement along pw3, camphor establishes a hydrogen bond with Thr-101. Camphor occupies the place previously taken by the side-chain of Tyr-96 in the proximity of Arg-299, which results in a local decrease of free energy (at σ ≈ 0.5). At this stage, The ligand is stabilized by the interaction with Thr-101 (Table S1). Next, the hydrogen bond with Thr-101 breaks, resulting in leaving the metastable state. At this point, camphor is in the vicinity of the heme propionate groups. Next, camphor reaches the proximal side of heme near Tyr-75 and Tyr-78 and migrates to solvent in the proximity of the C and L helices.

This pathway is noticeably shorter than the other pathways, which indicates that the number of transient hydrogen bonds between camphor and the aromatic side-chains along pw3 should be lower. In fact, this can be observed. On the other hand the rigidity of the heme group contributes to the free-energy barrier. The results show that the transport along pw3 is disfavored since the movement of camphor disrupts partially the hydrogen bonds between the heme propionate groups and Arg-299. Therefore, the tunnel leading from the active site to the proximal side of heme near the C and L helices is improbable for such a rigid and nonpolar ligand like camphor.

### Induced-fit mechanism

In cytochrome P450cam, the mechanism for substrate binding depends on the size of the ligand^32^. According to Markwick *et al*.^36, 37^ larger ligands known to interact with the enzyme ingress the cytochrome P450cam active site and trap the ligand-bound system in an open conformation via a population shift mechanism^38^. Small ligands (e.g., camphor) fully enter the binding pocket which causes the induced-fit mechanism, leading to the transition from the open to the thermodynamically-preferable closed state. Many X-ray structures of cytochrome P450cam in the ligand-bound state indicate that the conformational change triggered by the binding of substrates involves the movement of the H, G, F and B′ helices, which are in close proximity to the SRS^32^. The SRS is highly conserved among the cytochrome family, showing the importance of this region for functioning of the enzyme^39^, which includes the ligand diffusion within the enzymatic matrix. Our results indicate that the ligand diffusion pathway which engages in probing the SRS is pw2, and it is therefore crucial to investigate whether the conformations of the enzyme sampled along pw2 with metadynamics can be clustered into the open and closed states.

**Figure 3:**
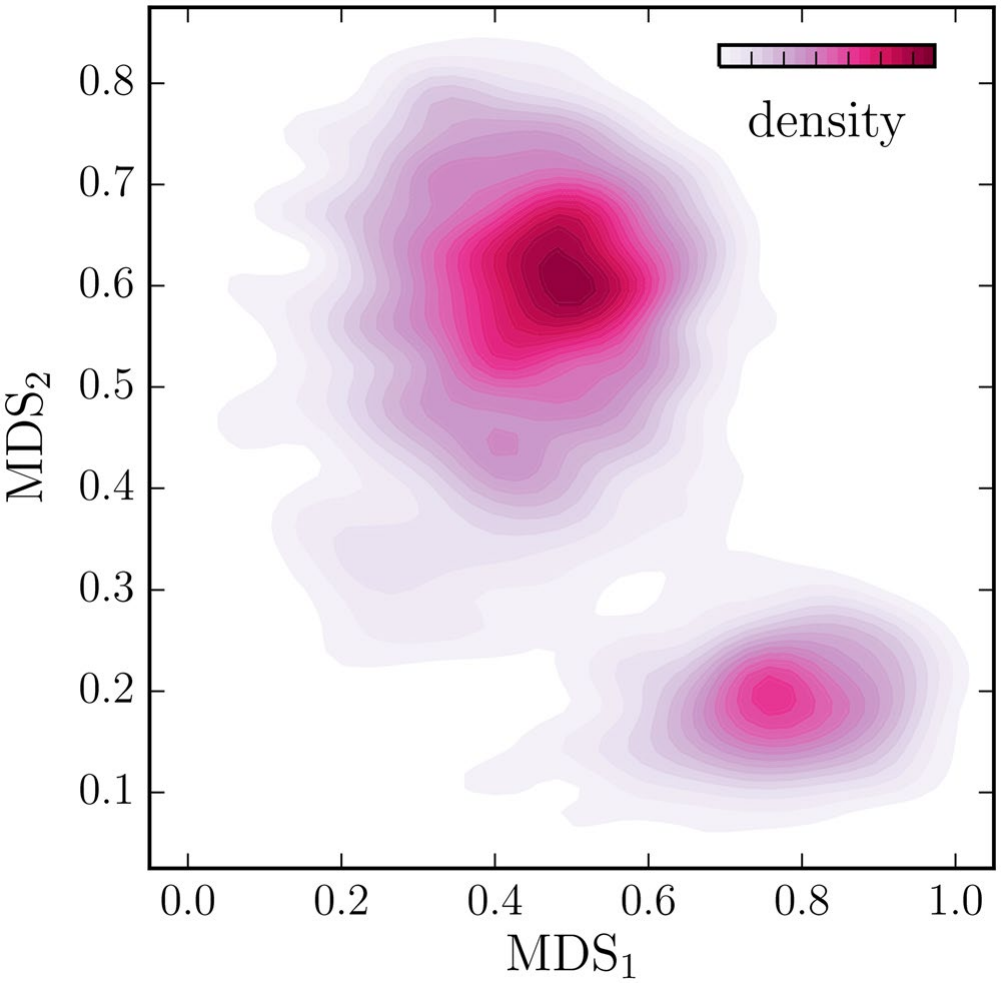
Conformational space of the camphor migration along pw2 in the proximity to the putative substrate recognition site of cytochrome P450cam retrieved using the multidimensional scaling. The conformations of the enzyme are split into the open ({0.5, 0.6}) and closed ({0.8, 0.2}) states. For the clustering of these conformers into the well-defined collections see Figs S2–S5 of the Supplementary Information.

The cytochrome P450cam C*α* atoms of the conformations sampled by metadynamics along the three accessible ligand diffusion pathways subjected to the multidimensional scaling (﻿MDS) indicate that the majority of the conformations occupy two basins (Fig. 3; for the full conformational space, we refer to Fig. S2 of the Supplementary Information). In order to assign the conformers to a particular basin, we performed clustering of the data embedded onto the two-dimensional space. As expected, our results show that the conformers can be separated into two states–open and closed. A majority of the conformations occupy the open basin (0.681) in comparison to the closed ligand-bound basin (0.213). For details concerning the DBSCAN clustering, see Figs S2–S5 in the Supplementary Information. The closed state is located at {0.8, 0.2} and the open state at {0.5, 0.6} as indicated in Fig. 3.

**Figure 4:**
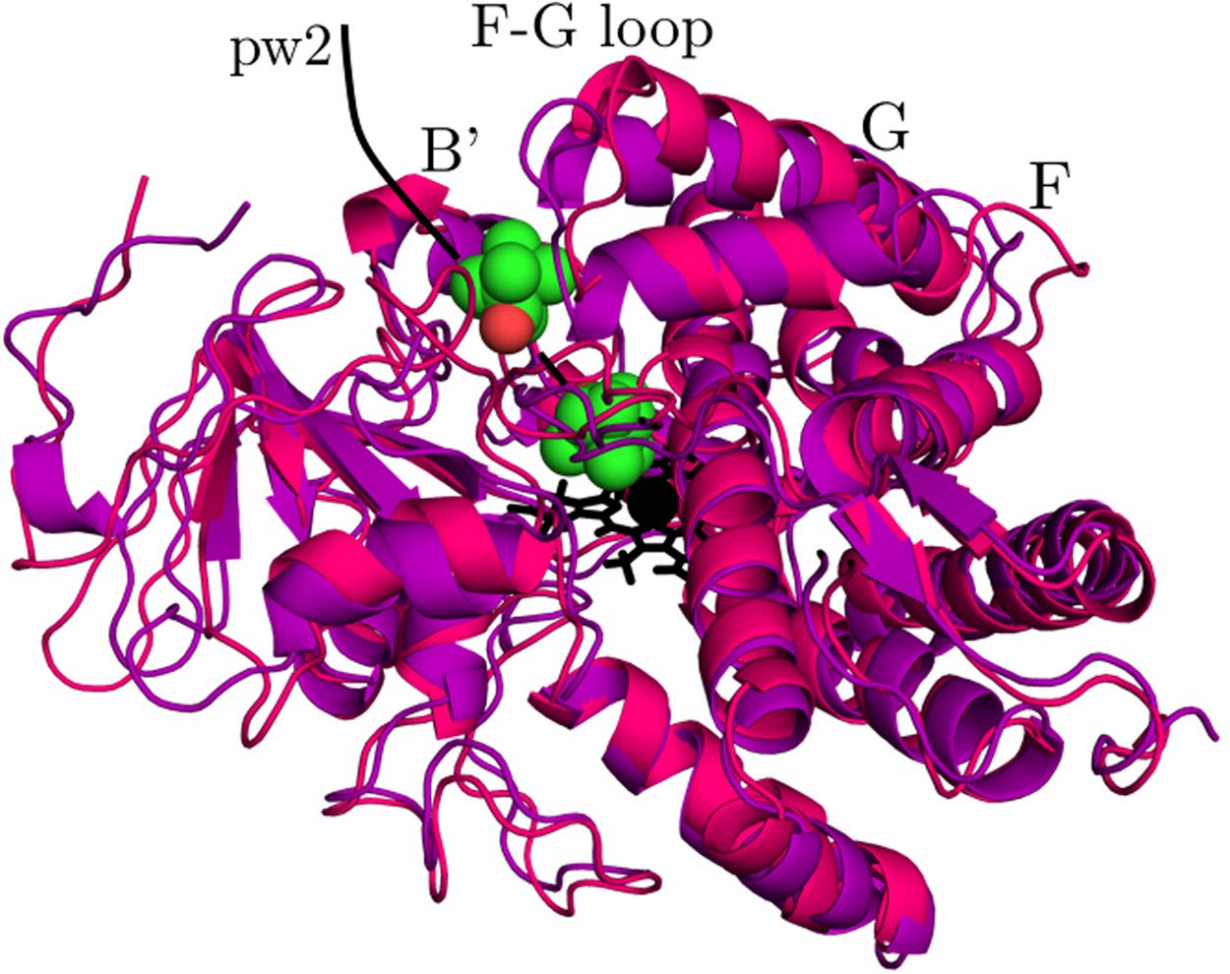
Representative conformations of cytochrome P450cam from the open and closed basins sampled during the diffusion along pw2 in the proximity of the putative substrate recognition site recovered using the multidimensional scaling. The open and closed conformations clustered into the open and closed states by DBSCAN are shown in pink and purple, respectively. The root-mean-square distance between these structures is 3.15 Å.

The metadynamics simulations start from probing the closed conformational basin in which camphor is bound within the enzymatic matrix. This basin is smaller than the open state basin, and the density of conformations is lower, suggesting that the diffusion of camphor within cytochrome P450cam is triggered by a jump of a trajectory from the closed to open state and its related conformational change. The closed basin is more structurally homogeneous comparing to the open basin, which comprises the need of a well-defined collection of conformers for the ligand-bound state. We show that the camphor binding selectively stabilizes the closed cytochrome P450cam conformers which agrees with IR vibrational echo spectroscopy experiments^40^. This result is in agreement with the analysis of the crystal structures of cytochrome P450cam^36^ and double electron-electron resonance study that demonstrates that the substrate-free enzyme prefers the open conformation and that the camphor binding results in conversion to the closed state^41, 42^. We observed that the main conformational change in the enzyme related to the diffusion of camphor along pw2 is an upward motion of the F, G and H helices, triggering salt-link tethering in close proximity of the SRS (Fig. 4) as shown previously using paramagnetic NMR spectroscopy^43^. Also the B′ helix undergoes an adjustment of its residues to the migrating camphor. We examined the X-ray structures of cytochrome P450cam with different ligands (PDB IDs: 1RE9, 1RF9, 3P6T, 3P6X) by calculating root-mean-square distance to conformations probed by the simulations. The X-ray structures fall into the closed state which is in agreement with a study of Markwick *et al*. and experimental studies^44^. Moreover, we investigated the X-ray structure of cytochrome P450cam with two camphor molecules bound in the open state conformation (PDB ID: 4JX1)^34^. This crystal structure falls into the open basin and depicts that a key feature in trapping a second camphor molecule in the pw2 exit route is a rotation of Tyr-96 in the opposite direction of the active site, which is also observed in our simulations.

We found that cytochrome P450cam exists in two well defined basins in the conformational space during the camphor diffusion, which agrees with the notion that the functional interplay between the enzyme conformers exists between the open and closed conformations due to the inducted-fit mechanism^38, 43^. Surprisingly, these basins are sampled only by the conformers neighboring the ligand migration along pw2. This shows the importance of pw2 in the diffusion of ligands and the functional motions of the enzyme indicating its molecular plasticity.

## Conclusions

In conclusion, we introduced a novel computational methodology for characterizing complex structural transitions of ligands in enzymes that allowed for atomic-level reconstruction of the reaction coordinates of ligand diffusion and free-energy profiles along these pathways. The detailed information provided by the performed simulations can be used to design new experiments, for instance, to test potential inhibitors of the cytochrome family that are suggested to alter drug metabolism for individuals on certain medications. We note that our methodology is general and can be applied to other ligand-protein complexes.

We presented an investigation directed toward uncovering the ligand diffusion mechanism of camphor in cytochrome P450cam during both biased and unbiased all-atom MD simulations in explicit solvent. To calculate the reaction coordinates describing transitions between the active site of cytochrome P450cam and three alternative exit routes we used a novel method, which sampled the conformations via biased MD simulations by minimizing effective interaction energy between camphor and cytochrome P450cam^23, 24^. The configuration space was explored by biasing pathways defined based on the previously identified reaction coordinates by the well-tempered metadynamics with the adaptive Gaussians, which rendered the free-energy profiles along the reaction coordinates in a quantitative manner.

The results of MD simulations of P450cam suggest that the diffusion of camphor along pw2 near the SRS is thermodynamically preferred over pw1 and pw3. The camphor diffusion along pw1 deviates the heme group and the I helix, and along pw3 disrupts the hydrogen bonds between the heme propionate groups and cytochrome P450cam. For these reasons the free-energy barriers along pw1 and pw3 are higher compared to free-energy values met along pw2. The egress mechanism along each pathway involves a ratcheting movement of camphor in the proximity of gating side chains of aromatic residues, by either passing or pausing camphor. Furthermore, the conformational flexibility of the active site in the crystallographic structure enables the adjustment of camphor to Tyr-96 resulting in stronger interactions between the active site and the camphor carbonyl oxygen.

We found that the migration near the SRS triggers a transition from a heterogeneous collection of closed ligand-bound conformers to the basin comprising of the open conformations of cytochrome P450cam. The presence of the two basins indicates that these two basins consist of the structures of cytochrome P450cam engaging in the induced-fit mechanism. Upon camphor binding the enzyme sinks into the closed basin which is a collection of heterogeneous conformers, leading to the protein zipping up around the bound substrate. The conformational change accompanying the switch between the closed and open conformers is characterized by the retraction of the F and G helices and the disorder of the B′ helix. These results are corroborated by experimental studies^40, 41, 44^ and potentially provide detailed insight into ligand migration and conformational behavior of the cytochrome family.

## Methods

### Metadynamics simulations

Before running metadynamics simulations the camphor-cytochrome P450cam complex (PDB ID: 2CPP)^33^ was minimized and equilibrated through 5 ns MD simulation under NPT conditions at 1 atm and 300 K (the velocity-rescaling thermostat^45^ and Berendsen’s barostat)^46^ using the CHARMM27 force field^47^ as implemented in Gromacs-5.0.6^48^. Standard parameters were used for camphor and the heme group^2, 49, 50^ including an explicit Fe-S bond between the heme group and Cys-357^51^. Point charges for camphor were collected from the article of Schöneboom *et al*.^52^. All the simulations were run using the TIP3P water model^53^ in electrically neutralized environment with periodic boundary conditions. The long-range electrostatics was computed by the particle mesh Ewald (PME) method^54^. Bonds were constrained holonomically by using the P-LINCS algorithm^55^. Three arbitrary C*α* atoms of cytochrome P450cam (Met-261, Trp-374, Ser-141) were harmonically restrained in each direction in order to prevent the enzyme from movement caused by the diffusing camphor. They were not a part of any conformational change during the diffusion process. To prohibit camphor from migrating from the cytochrome P450cam matrix to solvent, additional constraints for the distance between Fe and the center of mass of camphor were added for each exit pathway (Table 1).

**Table 1:** Reaction coordinates representing 3 pathways of the camphor diffusion from the cytochrome P450cam active site.

pathway	simulation time [*μ*s]	no. of frames	〈MSD〉^a^[Å^2^]	*λ*[Å^−2^]	*d* ^b^[Å]	〈SE〉^c^ [kcal/mol]
pw1	0.30	11	14.41	0.16	21	0.2
pw2	0.20	9	13.03	0.18	26	0.1
pw3	0.25	11	6.31	0.36	15	0.1

^a^Mean-square deviation of the displacement set.

^b^Distance between the center of mass of camphor and Fe.

^c^Mean standard error of free energy along the pathway after 100 ns of the simulation.

The time-independent estimator of free energy *F*(*ξ*) at time *t* of the free-energy profiles *F*(*ξ, t*) as a function of CVs *ξ* was determined by metadynamics^56^ in the well-tempered variant^57^ using $F(\xi ,t)=-\frac{T+{\rm{\Delta }}T}{{\rm{\Delta }}T}V(\xi ,t)$, where $V(\xi ,t)$ is the biasing potential added to the system and *T* is the temperature used in the simulation. The difference between the temperature of the CV and the temperature of the simulation is denoted by Δ*T*. The bias potential is made by the sum of the Gaussians deposited along the trajectories of the CVs, *ξ*(*t*). We also used the adaptive Gaussians^58^. The free-energy profiles were reweighted by the time-dependent constant *c*(*t*):

1 $$c(t)=\frac{1}{\beta }ln\frac{\int d\xi {e}^{-\beta F(\xi )}}{\int d\xi {e}^{-\beta [F(\xi )+V(\xi ,t)]}}$$

where *β* is the inverse temperature (1/*k*_*B*_ *T*), in order to achieve the time-independent estimator of free energy^59^.

For a detailed description of metadynamics, we refer to several reviews^60, 61^. Metadynamics simulations were carried out in NVT ensemble using the PLUMED2^62^. During the course of metadynamics simulations, we used the following parameters. The adaptive Gaussians were set to be in the time domain and the width of the Gaussian was set to 0.25 ps. The rate of the Gaussian deposition was 1.92 kcal/mol per ps. The temperature in the well-tempered protocol was 300 K and the Δ*T* was 3300 K.

The time-independent estimator of free energy was used to ascertain the local quality of convergence across the collective variable space^59^. Additionally, the convergence of the free-energy calculations was monitored by plotting the Gaussian height as a function of the simulation time. The free energy was considered partially converged when the Gaussian height decreased to zero^57^. Moreover, the full convergence required multiple recrossings of *ξ* during which the diffusion pathways were sampled several times in the collective variables space. The residual height of the Gaussian was less than 1% of the initial height after the simulation for each exit route. Therefore, the convergence of the free-energy profiles reweighted by *c*(*T*) (cf. Methods) was stated for those simulations in which the residual height was less than 1% of the initial height and no further refinement of the reweigthed free-energy profiles was observed. The standard error of free energy along the diffusion pathways after 100 ns was also calculated (Fig. S6 in the Supplementary Information).

Conformations sampled during the metadynamics simulations (C*α* atoms) were subjected to the multidimensional scaling^63^ (MDS) to embed the multidimensional simulation data into a 2-dimensional space and subsequently clustered using DBSCAN (density-based spatial clustering of applications with noise), which is an algorithm used to cluster data with noise based on densities^64^. For details including the specific selection of input parameters, please see Figs S2–S5 of the Supporting Information.

### Reaction coordinates

Each diffusion pathway is atomistically characterized by the reaction coordinate that consists of conformations *S*(1), …, *S*(*N*), where *N* is the number of conformations describing the transition between the bound and unbound states, and *S* is a conformation of a ligand-protein complex. Each reaction coordinate was computed in our previous article^24^ (Fig. S1) by means of MS^23^. In MS, a biasing force is used to guide the ligand along the diffusion pathway, toward the *k*th conformation (Fig. S7). The biasing force is maintained during *m* steps of MD. The *k*th conformation is determined on-the-fly by minimizing the functional Λ which describes effective interaction energy between the protein and ligand. We used Λ of the following form:

2 $${\rm{\Lambda }}=\sum _{i < j}{h}_{ij}{{\rm{e}}}^{-\frac{{r}_{ij}^{2}}{2{w}^{2}}}+\gamma ,$$

where *i* and *j* indicate atoms indices of the ligand and the protein, respectively. In Eq. 2 the distance between the atoms is denoted by *r*_*ij*_. The height of the Gaussian is defined as ${h}_{ij}={s}_{i}{v}_{j}+{s}_{j}{v}_{i}$, where *s* and *v* are the partial volume and the solvation coefficient of the given atoms. The width of the Gaussian is given by *w*. Additionally, we added the term *γ* describing the van der Waals interactions and electrostatics between the ligand and protein to Λ. The algorithm for finding *k*th conformation *S*(*k*) (given *S*(*k* − 1) is known) involves the following steps:

1. The *k*th ligand conformation in a local neighborhood of the (*k* − 1)th ligand conformation is found by minimizing Λ. [The local domain is assured by sampling the ligand conformations inside a sampling sphere centered at the (*k* − 1)th conformation];
2. The ligand is pulled by the biasing force in *m* steps of the MD simulation in the direction of the *k*th ligand conformation;
3. After *m* steps of an MD simulation, the conformation of the system consisting of the *k*th ligand conformation and the protein that is the course of the MD simulation adjusted to the moving ligand, is saved as the *k*th conformation *S*(*k*).

Details concerning minimizing the functional Λ can be found in refs 23, 24. The conformations defining the three exit routes of camphor (Fig. S1) were used to perform metadynamics simulations in a path collective variable (PCV) space.

### Path collective variables

The camphor diffusion pathways in cytochrome P450cam were studied using PCVs^65^. These pathways were represented in the space of the mean-square deviation of the camphor and P450cam coordinates. PCVs require two sets of atoms for the metadynamics simulations. The alignment set was used to superimpose the reaction coordinates with the current state of the system, whereas the displacement set is employed to compute the mean-square displacement between the aligned conformations. The sets for alignment and displacement consisted of the C*α* atoms of the I, G and C helices, and camphor, respectively. These sets were modeled after on previous calculations of the ligand diffusion^20, 66, 67^.

The reactions coordinates describe 3 pathways between the bound and unbound states. To follow these pathways, *s*(**R**) and *z*(**R**) PCVs were used^20, 65^:

3 $$s({\bf{R}})=\frac{\sum _{k\mathrm{=1}}^{N}k{{\rm{e}}}^{-\lambda \parallel S({\bf{R}})-S(k){\parallel }^{2}}}{\sum _{k\mathrm{=1}}^{N}{{\rm{e}}}^{-\lambda \parallel S({\bf{R}})-S(k){\parallel }^{2}}}$$

and

4 $$z({\bf{R}})=-\frac{1}{\lambda }\,\mathrm{ln}\,\sum _{k\mathrm{=1}}^{N}{{\rm{e}}}^{-\lambda \parallel S({\bf{R}})-S(k){\parallel }^{2}},$$

where *S*(**R**) is a reduced representation of a configuration **R**. The progress along the reaction coordinate is computed by *s*(**R**); the distance (deviation) from the reaction coordinate is measured by *z*(**R**). The *λ* values for each reaction coordinate are shown in Table 1. Typically, with a reasonable guess of the reaction coordinate values of *z*(**R**) should be small which means that the system does not deviate much from the reaction coordinate. We performed metadynamics only in the space of *s*(**R**) while *z*(**R**) was constrained to $z({\bf{R}}) < 6{{\rm{\AA }}}^{2}$. This choice gives the possibility for the system to explore conformations different from *S*, however maintaining at the same time the system reasonably close to the chosen reaction coordinates.

## Electronic supplementary material

supplement
