# Supplementary material for: Thermodynamics of camphor migration in cytochrome P450cam by atomistic simulations
Source: Sci Rep. 2017 Aug 10;7:7736. doi: 10.1038/s41598-017-07993-0 (PMC5552751; doi:10.1038/s41598-017-07993-0)
Supplement: Supplementary file 1 — supplement [file 41598_2017_7993_MOESM1_ESM.pdf]

Thermodynamics of camphor migration in cytochrome P450cam by atomistic simulations

Supporting Information

J. Rydzewski[1,*] and W. Nowak[1]

[1]*Institute of Physics, Faculty of Physics, Astronomy and Informatics, Nicolaus Copernicus University, Grudziadzka 5, 87-100 Torun, Poland*

(Dated: June 20, 2017)

I. CAMPHOR EGRESS TRAJECTORIES

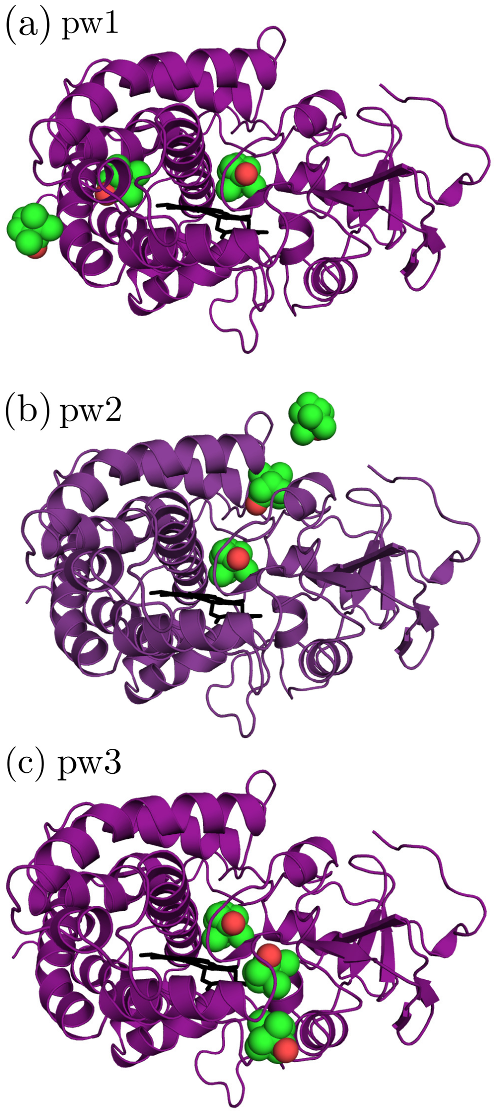

FIG. S1. Representative trajectories of the camphor escaping from the buried cavity of cytochrome P450cam via pw1-3 used to define the reaction coordinates of the camphor diffusion, calculate the collective variables and run metadynamics simulations. The enzyme and the heme group are colored in purple and black, respectively. Camphor is represented by spheres in green (carbon) and red (oxygen).

TABLE S1. List of the cytochrome P450cam residues occurring along the diffusion pathways pw1-3 in close proximity (< 4Å) of camphor at the given values of $s(\boldsymbol{R})$ and σ.

pathway	$s(\boldsymbol{R})$	σ	residues
pw1	1	0.0	Thr-185 Leu-244 Val-247 Gly-248 Thr-252 Val-295 Ile-395 Val-396 Phe-87 Tyr-96
	2	0.1	Leu-244 Val-247 Gly-248 Gly-249 Thr-252 Tyr-96
	3	0.2	Ala-115 Val-223 Met-241 Cys-242 Leu-245 Leu-246 Leu-358
	4	0.3	Ile-220 Cys-242 Leu-245 Leu-246 Gly-249 Leu-250
	5	0.4	Val-118 Val-119 Leu-166 Ala-219 Leu-245 Leu-362
	6	0.5	Pro-159 Ile-162 Phe-163 Leu-166 Ala-219 Ile-220
	7	0.6	Val-123 Lys-126 Leu-127 Arg-130 Ile-162 Leu-166
	8	0.7	Val-123 Lys-126 Leu-127 Arg-130 Ile-162 Leu-166 Ala-219
	9	0.8	Lys-126 Leu-127 Arg-130 Leu-134 Ile-162 Leu-165 Leu-166
	10	0.9	Lys-126 Arg-130 Leu-134 Ile-162 Leu-165 Leu-166
	11	1.0	Lys-126 Leu-127 Asn-129 Arg-130 Leu-166 Thr-217
pw2	1	0.0	Thr-185 Leu-244 Val-247 Gly-248 Thr-252 Val-295 Ile-395 Val-396 Phe-87 Tyr-96
	2	0.125	Thr-181 Met-184 Thr-185 Phe-193 Leu-244 Val-247 Gly-248 Ile-395 Val-396 Phe-87 Tyr-96
	3	0.25	Thr-181 Met-184 Thr-185 Phe-193 Val-247 Ile-395 Val-396 Phe-87 Tyr-96
	4	0.375	Met-184 Thr-185 Phe-193 Ile-395 Val-396 Phe-87 Tyr-96
	5	0.5	Met-184 Thr-185 Phe-193 Val-247 Ile-395 Phe-87 Ala-92 Gly-93 Phe-98
	6	0.625	Thr-185 Pro-187 Phe-193 Tyr-29 Ile-395 Phe-87 Ala-92
	7	0.85	Pro-187 Ala-29 Ile-395 Phe-87 Ile-88 Pro-89 Ala-92 Tyr-96
	8	0.975	Pro-187 Ala-29 Phe-89 Glu-91 Ala-92
	9	1.0	Pro-187 Thr-192 Pro-89 Glu-91 Ala-92
pw3	1	0.0	Thr-185 Leu-244 Val-247 Gly-248 Thr-252 Val-295 Ile-395 Val-396 Phe-87 Tyr-96
	2	0.1	Thr-101 Leu-244 Tyr-96 Phe-98
	3	0.2	Thr-101 Phe-193 Leu-244 Val-247 Ile-395 Pro-86 Phe-87 Tyr-96 Phe-98
	4	0.3	Thr-101 Leu-244 Phe-87 Tyr-96 Phe-98
	5	0.4	Thr-101 Ser-102 Leu-244 Pro-86 Phe-87 Tyr-96 Asp-97 Phe-98
	6	0.5	Thr-101 Leu-244 Arg-299 Ser-83 Pro-86 Phe-87
	7	0.6	Thr-101 Arg-299 Ser-83
	8	0.7	Gln-108 Arg-299 Tyr-75 Tyr-78
	9	0.8	Gln-108 Arg-299 Tyr-78 Ser-83
	10	0.9	Tyr-75 Tyr-78 Ser-83
	11	1.0	Leu-356 Tyr-75 Glu-76 Tyr-78

II. MULTIDIMENSIONAL SCALING

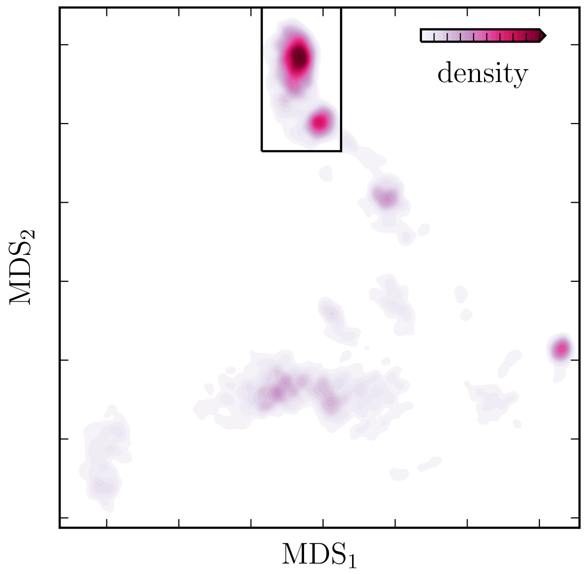

FIG. S2. Conformational space of the camphor migration along pw1-3 of cytochrome P450cam retrieved using the multidimensional scaling. The selected (by frame) conformations of the enzyme indicate the camphor diffusion along pw2 and the substrate recognition site. For the conformation space probed during diffusion along pw2, see Fig. 3 in the main text.

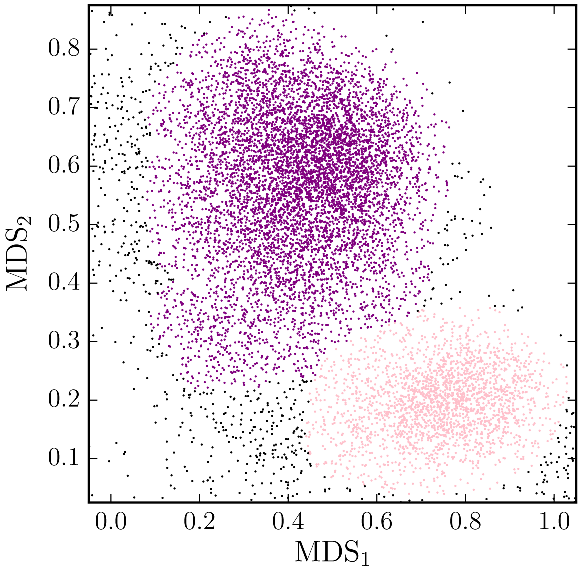

FIG. S3. Clustering of the conformational space of the camphor escape along pw2 using DBSCAN. The algorithm found two clusters consisting of the open state (with camphor unbound) and the closed state (with camphor bound); shown in purple and pink, respectively. The conformers depicted by black dots were labeled as noise data. This clustering is done for the following clustering radius $r = 0.25$ and the minimal number of conformers within a cluster $m = 100$, selected by maximizing the silhouette score s which provides the maximal fraction of conformers clustered into states and the minimal fraction of noise. The input results in $s = 0.47$.

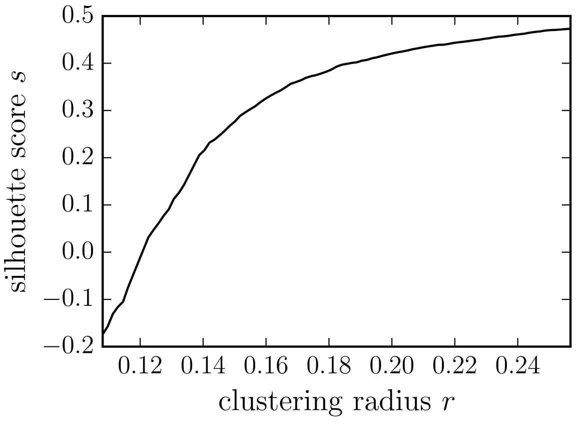

FIG. S4. Silhouette score values for the increasing radius r of clusters used to establish the input data for DBSCAN. The clustering radius $r = 0.25$ shows a sufficient silhouette for clustering. [The silhouette value is a measure used to determine how similar an object is to its own cluster (cohesion) compared to other clusters (separation). The silhouette score ranges from -1 to 1, where a high value indicates that the object is well matched to its own cluster and poorly matched to neighboring clusters.]

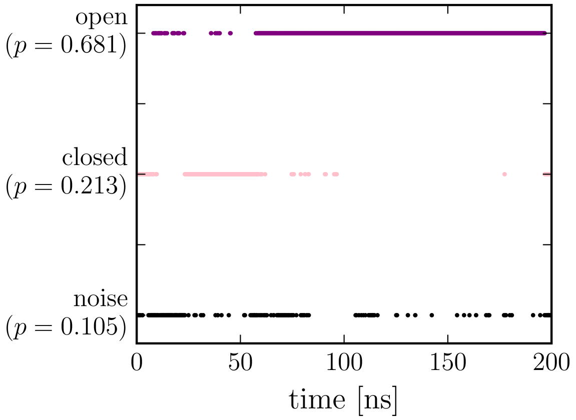

FIG. S5. Labeling of conformers during the diffusion of camphor along pw2. The states (open, closed and noise) were defined by the DBSCAN clustering, see Figs. S3 and S4. The fractions p of sampling a conformation in the given states are noted.

III. FREE ENERGY

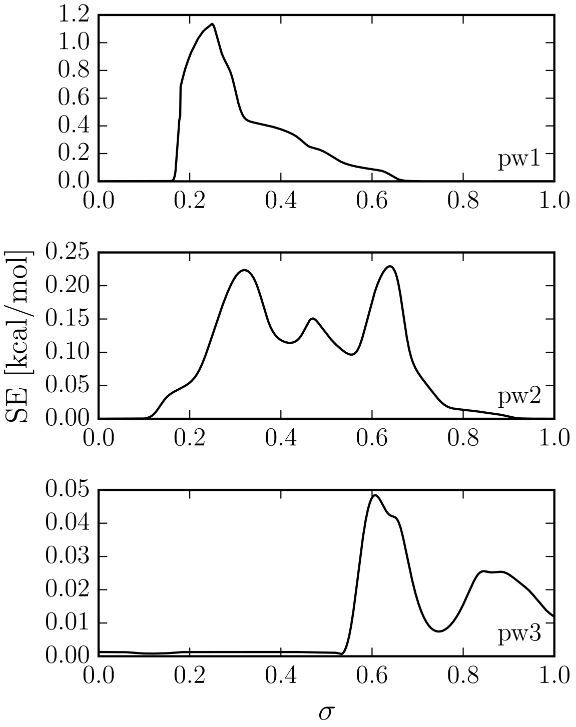

FIG. S6. Standard error of free-energy profiles $F(\sigma)$ along the camphor diffusion pathways pw1-3 calculated by observing incremental additions in free energy after 100 ns of metadynamics simulations. [The order parameter σ is introduced by scaling s.]

TABLE S2. List of the cytochrome P450cam local minima and maxima occurring along the diffusion pathways pw1-3. [For each minimum a 10 ns unbiased molecular dynamics simulation was run; for each maximum–50 short unbiased molecular dynamics simulations.]

pathway	starting s	starting σ	$s(\boldsymbol{R})$	σ	no. of trajectories	time
pw1	1.13	0.113	1.18±0.09	0.017±0.008	1	10 ns
	3.01	0.201	1.12±0.09	0.012±0.009	1	10 ns
	4.99	0.299	5.52±0.52	0.451±0.051	1	10 ns
	6.01	0.501	4.99±0.19	0.399±0.019	1	10 ns
	2.86	0.186			50	<473 ps
	3.72	0.272			50	<154 ps
	5.63	0.463			50	<3 ps
	7.58	0.658			50	<840 ps
pw2	1.13	0.016	1.24±0.09	0.031±0.012	1	10 ns
	3.34	0.293	1.20±0.11	0.022±0.012	1	10 ns
	7.87	0.856	8.74±0.53	0.860±0.059	1	5 ns
	1.99	0.124			50	<37 ps
	5.52	0.565			50	<81 ps
	7.78	0.848			50	<578 ps
pw3	1.10	0.010	1.20±0.16	0.020±0.016	1	10 ns
	4.36	0.336			50	<1 ps
	5.61	0.461			50	<12 ps
	7.50	0.650			50	<268 ps
	9.54	0.854			50	<58 ps

IV. OPTIMALITY OF THE REACTION COORDINATES

The reaction coordinates for the camphor egress pathways have been identified by a variant of steered MD in which an external force is applied to the ligand in order to sample its transition from the active site to solvent (cf. Reaction coordinates). Nevertheless, several aspects of the optimality of the reaction coordinates require discussion. Namely, if a determined reaction coordinate is not optimal, it is a common procedure to allow the system to explore alternative pathways in $\boldsymbol{\xi}$ by increasing the accessible distance from the reaction coordinate (by constraining the upper wall on $z(\mathbf{R})$). In such instances, by biasing also $z(\mathbf{R})$ the system is able to refine the reaction coordinate and if it is not chosen optimally, metadynamics will find the minimum free-energy path. But, since the reaction coordinates were determined optimally by minimizing interaction free-energy Λ, only $s(\mathbf{R})$ was biased in our metadynamics simulations. All pathways were constrained to $z(\mathbf{R}) < 6$Å in order to allow for the system local fluctuations when camphor was in close proximity of the regions

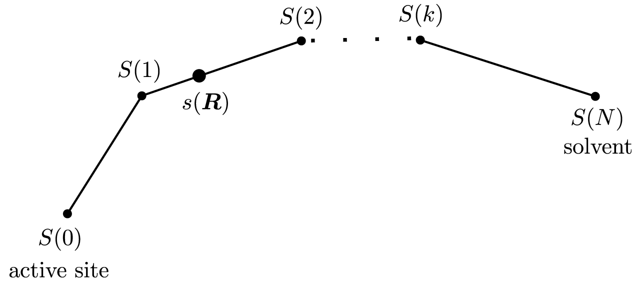

FIG. S7. Sketch showing how the reaction coordinate describing the camphor diffusion from cytochrome P450cam through intermediate binding sites is determined. Conformations $S(1), \ldots, S(N)$ are identified by calculating a local minimum of the functional Λ quantifying interactions between the ligand and protein. First, $S(1)$ is reached in m steps of a MD simulation. Therefore, $S(1)$ corresponds to the conformation of the system consisting of the ligand position previously found by minimizing Λ and the protein conformation at the time of reaching the Λ minimum. The protein conformation adjusts itself to the replacement of the ligand. Next, after the first conformation is passed, $s(\mathbf{R})$ moves toward $S(2)$ which is also determined by minimizing Λ, but in a local neighborhood of $S(1)$. The algorithm is repeated until the exit route in the protein tunnel is calculated.

of low thermal motions in cytochrome P450cam (i.e., the I helix on pw1).

The search for a reaction coordinate in the ligand diffusion process is surely approximate, because it depends on the choice of the functional quantifying physical interactions between the protein and ligand. Elber and Gibson argued in the context of locally enhanced sampling performed for myoglobin that such searches tend to overestimate preference to more direct and geometrically short paths even if they are energetically costly. Following this observation, our results indicated that it is not true for our reaction coordinates. For instance, the highest free-energy barrier can be noticed along pw3 which is also the shortest diffusion route, and the number of trajectories sampled on this pathway is not dominant. Moreover, the most preferred pathway (pw2) is the longest one. Therefore, unlike in locally enhanced sampling, the optimality of the reaction coordinates is not compromised, making it possible to bias only $s(\mathbf{R})$ and to put the upper wall on $z(\mathbf{R})$ close to the reaction coordinates.
